# Rapid and Non-Destructive Detection of Moisture Content in Dried Areca Nuts Based on Near-Infrared Spectroscopy Combined with Machine Learning

**DOI:** 10.3390/foods15081359

**Published:** 2026-04-14

**Authors:** Jiahui Dai, Shiping Wang, Xin Gan, Yanan Wang, Wenting Dai, Xiaoning Kang, Ling-Yan Su

**Affiliations:** 1 College of Food Science and Technology, Yunnan Agricultural University, No. 452 Fengyuan Road, Kunming 650000, China; jiahui_dai@163.com (J.D.);; 2 Haikou Areca Processing Research Key Laboratory, Institute of Agro-Products Processing and Design, Hainan Academy of Agricultural Sciences, Haikou 571100, China; 3 Sinolight Inspection and Certification Co., Ltd., Beijing 100000, China

**Keywords:** areca nut, moisture content, near-infrared spectroscopy, non-destructive detection, machine learning

## Abstract

Moisture content is a key quality attribute in dried areca nuts, affecting subsequent processing performance and storage stability, yet routine measurement by oven-drying is time-consuming and destructive. This study developed a rapid and non-destructive method for determining moisture content in dried areca nuts by integrating near-infrared spectroscopy with chemometric and machine learning-assisted methodologies. Various spectral preprocessing methods, feature wavelength selection algorithms, and modeling approaches were compared. The results indicated that Multiplicative Scatter Correction (MSC) most effectively eliminated physical scattering interference. The Partial Least Squares Regression (PLSR) model established using full-wavelength spectra demonstrated optimal predictive performance. It achieved a coefficient of determination for the prediction set (Rp^2^), root mean square error of prediction (RMSEP), and residual predictive deviation (RPD) of 0.9639, 0.1960, and 10.3461, respectively, indicating excellent predictive accuracy and robustness. Feature wavelength selection did not enhance model performance in this study, which can be attributed to the broad absorption bands of water in the near-infrared spectrum and its complex interactions with the sample matrix where the full spectrum data retains essential information more comprehensively. This research provides a reliable and practical technical means for moisture management in areca nuts, offering important support for quality assurance and standardized production practices within the areca industry.

## 1. Introduction

Areca nut, the seed of the palm *Areca catechu* L., is one of the most consumed psychoactive substances in the world, with hundreds of millions of consumers across Asia, East Africa, and the Pacific region [1,2]. In China, its consumption primarily involves chewing it as a processed stimulant [3]. The processing of areca nuts consists of two main stages: primary processing and deep processing [4,5,6]. The primary processing phase includes boiling freshly harvested nuts in hot water to inactivate enzymes, followed by oven roasting to reduce moisture content to approximately 15–20% [4]. This initial processing phase serves two critical functions: Firstly, as the foundational product stage, it critically influences the quality of subsequent deep processed products; moisture content that is either too high or too low adversely affects the final texture and chewiness. Secondly, it mitigates quality issues such as mold growth during storage and transport [7,8]. For manufacturers engaged in deep processing, fluctuations in moisture content are directly linked to production expenses. Consequently, accurate assessment of moisture content in dried areca nuts is essential for ensuring product quality and controlling production costs [9,10].

Traditional moisture determination methods, such as the oven direct drying method, require drying the weighing bottle (1.5 h) prior to placing the sample in the bottle and drying to a constant weight in an oven (approximately 4–6 h in total). This process is extremely time-consuming and consumes a significant amount of electrical energy, limiting its applicability for high-throughput online or in-process quality control in large-scale production. This creates a practical need for rapid, non-destructive analytical methods that can be deployed routinely on the factory floor.

Near-infrared spectroscopy (NIRS) is widely regarded as a rapid, non-destructive, and environmentally friendly analytical technique for assessing the quality of agricultural products [11,12,13,14,15]. By capturing absorption, reflectance, and scattering responses in the near-infrared region, NIRS can provide rich information related to key constituents such as moisture, protein, and fat, thereby enabling fast quality evaluation [16,17,18]. However, NIR spectra are typically characterized by broad and overlapping bands, and are easily affected by physical factors such as particle size, surface texture, and path-length variation, which can introduce scattering and baseline distortions. As a result, extracting quantitative relationships between spectra and target properties usually requires data-driven modeling (i.e., chemometrics/machine learning) to denoise spectra, correct interferences, and learn robust calibration functions from reference measurements. Consistent with this perspective, previous studies have demonstrated NIRS-based moisture quantification in multiple commodities, including tea, coffee beans, corn, and honey. For example, Liu et al. [19] improved VIS–NIR moisture prediction for tea leaves via transfer learning to enhance model generalization across conditions; Ordoñez-Lozano et al. [20] monitored moisture in parchment coffee beans during drying using FT-NIR and provided a dataset to support chemometric model calibration; Li et al. [21] proposed a moisture-interference correction method to improve the reliability of maize powder NIR analysis; and Biswas and Chaudhari [22] reviewed NIR applications in honey, highlighting its utility for rapid quantification and verification in food quality assessment. These studies collectively underscore that combining NIRS with chemometric/machine learning models is essential for achieving accurate and robust moisture prediction in complex agricultural matrices.

In recent years, near-infrared spectroscopy has demonstrated broad application potential for quality assessment in a wider range of agricultural products. For instance, Zhong et al. [15] combined NIR spectroscopy with feature variable selection to predict key volatile organic compounds in Gastrodia elata processed under different conditions. Huang et al. [11] employed visible and near-infrared spectroscopy coupled with deep learning to achieve rapid identification of early bruising in blueberries. Malavi et al. [23] integrated NIR hyperspectral imaging with machine learning algorithms to successfully detect adulteration of extra-virgin olive oil with lower-grade oils. These studies further confirm the feasibility of NIR techniques for rapid, non-destructive analysis in complex food matrices.

However, several research gaps remain. First, most existing studies have focused on homogenized samples (e.g., powders or extracts), whereas the effects of surface heterogeneity and complex light scattering in intact granular samples on model robustness have not been sufficiently addressed. Second, for dried areca nuts, no study has systematically compared the performance of a chemometric method (PLSR) with that of a machine learning method (SVR) for moisture detection, leaving the linear versus nonlinear nature of the spectral data underexplored. Existing NIRS research on areca nuts has only focused on the rapid analysis of their active components [24]. Therefore, applying NIRS for moisture content determination in dried areca nuts can not only overcome the limitations of traditional methods, but also improve detection efficiency and reduce costs, which is of great significance for ensuring the quality of areca nut products.

This study aims to develop a rapid and non-destructive method for detecting moisture content in dried areca nuts based on NIRS technology. Near-infrared spectra were collected from samples of dried areca nuts. To enhance data quality, spectral preprocessing methods such as Standard Normal Variate (SNV), Multiplicative Scatter Correction (MSC), and Savitzky–Golay Smoothing (SGS) were employed to eliminate noise and scattering artifacts, in conjunction with reference moisture content measurements. A comparative analysis was conducted on the efficacy of the Successive Projections Algorithm (SPA) and Competitive Adaptive Reweighted Sampling (CARS) for selecting characteristic wavelengths. Multivariate statistical analysis methods, namely Partial Least Squares Regression (PLSR) and Support Vector Regression (SVR), were utilized to establish prediction models. The predictive accuracy, robustness, and practicality of the models were comprehensively evaluated using the coefficient of determination (R^2^), root mean square error of calibration (RMSEC), and root mean square error of prediction (RMSEP). This research provides a reliable and practical technical means for moisture management in areca nuts. Importantly, this study establishes the first validated near-infrared spectral dataset for intact dried areca nuts, comprising 300 samples with a moisture content range of 8.38–18.20%, which can serve as a benchmark for future non-destructive quality assessment studies. This method is applicable not only to moisture detection in dried areca nuts but can also serve as a reference for quality detection in other similar agricultural products, promoting the broader application of NIRS technology in this field. Furthermore, this research is crucial for enhancing quality control processes for areca nut products, safeguarding consumer health and safety, and promoting the sustainable growth within the areca nut industry.

## 2. Materials and Methods

### 2.1. Materials and Reagents

The dried areca nut samples used in this experiment were purchased from the Wanning Areca Nut Trading Market in Hainan Province, China. A total of 300 dried nut samples were collected.

### 2.2. Instruments and Equipment

A DGG-9140A Electric heating constant temperature air circulation drying oven (Donglu Instrument Equipments Co., Ltd., Tianjin, China), Frontier Fourier-Transform Infrared Spectrometer (PerkinElmer Inc., Waltham, MA, USA), and AP135W Electronic Balance (Shimadzu Corporation, Kyoto, Japan).

### 2.3. Determination and Calculation of Moisture Content in Dried Areca Nuts

The moisture content was determined according to the direct drying method specified in the Chinese National Standard GB 5009.3-2016. First, the samples were ground into powder and sieved through a 100-mesh sieve. Approximately 5 g of the powder was accurately weighed and placed in a drying oven. During the drying process, the sample was periodically removed and weighed until the mass difference between two consecutive weighings was no more than 2 mg, indicating that a constant weight had been achieved. The moisture content of the sample was calculated using the following formula:

(1) $$\mathrm{Moisture}\;\mathrm{Content}=\frac{\mathrm{Quality}\;\mathrm{before}\;\mathrm{drying}-\mathrm{Quality}\;\mathrm{after}\;\mathrm{drying}}{\mathrm{Quality}\;\mathrm{before}\;\mathrm{drying}}\times 100\%$$

To evaluate the measurement uncertainty of the reference method, 10 samples were randomly selected, and each sample was independently measured three times. The standard deviation of the three measurements was calculated for each sample, and the pooled standard deviation was then determined. The expanded uncertainty (U) was calculated using a coverage factor of k = 2, corresponding to a confidence level of approximately 95%.

### 2.4. Collection and Preprocessing of Near-Infrared Spectra

Spectra of the samples were collected at room temperature using a PerkinElmer NIRA II integrating sphere spectrometer. Three spectra were acquired for each sample by scanning three randomly selected points on the equatorial line of the areca nut using an infrared probe. The spectral acquisition parameters were set as follows: spectral range of 4000 to 10,000 cm^−1^, resolution of 8 cm^−1^, data spacing of 2 cm^−1^, 64 scans per spectrum, and a path length of 30 cm^−1^. Background scans were performed after every 12 sample scans to correct for instrumental drift. For subsequent modeling analysis, all collected spectral data were processed, and the three spectra per sample were averaged.

Different preprocessing algorithms were applied to the averaged spectra using MATLAB to enhance the signal quality. These included baseline correction using adaptive iteratively reweighted Penalized Least Squares (airPLS), normalization, Standard Normal Variate (SNV), Multiplicative Scatter Correction (MSC), and Savitzky–Golay Smoothing (SGS). These preprocessing steps effectively removed baseline and background interference and mitigated the effects of light scattering in the measurements [25].

### 2.5. Selection of Characteristic Wavelengths

Modeling using full wavelength spectral data often results in high model complexity, substantial computational load, and extended training times. Furthermore, the inclusion of irrelevant noise signals and redundant information can adversely affect the model’s predictive performance [26]. Therefore, prior to model establishment, employing suitable methods for the optimal selection of characteristic wavelengths to screen spectral variables is an indispensable and critical step in spectral analysis [27]. This study compared two feature wavelength extraction methods: Competitive Adaptive Reweighted Sampling (CARS) and the Successive Projections Algorithm (SPA). CARS is a feature wavelength selection method that combines Monte Carlo random sampling with PLS regression coefficients. This approach utilizes an exponentially decreasing function and adaptive reweighted sampling. After n sampling runs, the wavelength subset yielding the smallest Root Mean Square Error of Cross Validation (RMSECV) is selected [28]. SPA operates by screening the vast number of wavelength variables in the full spectrum to identify a minimal set of “characteristic wavelengths” that are highly correlated with the target property (moisture content). This serves to reduce model dimensionality, accelerate computations, and prevent overfitting [29].

To evaluate the stability of the feature selection results, the CARS algorithm was executed with 100 Monte Carlo sampling runs. The frequency of each wavelength being selected across these runs was recorded. The wavelengths selected in the optimal run (corresponding to the minimum RMSECV) were considered as the final feature set. Their average selection frequency was calculated to assess reproducibility. For SPA, the stability was evaluated through 5-fold cross-validation on the selected wavelengths.

### 2.6. Model Development and Validation

The preprocessed and characteristic wavelengths selected spectral data were used to establish prediction models for the moisture content of dried areca nuts by employing Partial Least Squares Regression (PLSR) and Support Vector Regression (SVR). The SVR algorithm was implemented using the Li-SVMLAB toolbox in MATLAB 2024b software. The radial basis function (RBF) was selected as the kernel function, and the Tunelssvm cross-validation function was employed to optimize the penalty factor (c) and kernel function parameter (g). The specific parameters are as shown in Table 1.

PLSR is one of the most widely used analytical methods in spectral analysis. It projects high dimensional spectral data onto a lower dimensional space of orthogonal latent variables by establishing a latent variable space between the spectral matrix and the target property matrix. Its core principle lies in extracting a sequence of latent variables that best explain the target property through covariance maximization decomposition [30]. SVR, based on the structural risk minimization principle of statistical learning theory, utilizes kernel functions to map nonlinear problems into a high-dimensional feature space. It optimizes regression by constructing an ɛ-insensitive band, applying a penalty only to samples falling outside this tolerance zone [23]. Both methods have distinct advantages. PLSR is characterized by its intuitive principles, computational efficiency, and relatively good model interpretability, making it suitable for datasets with strong linear relationships. In contrast, SVR is better at capturing complex nonlinear relationships and is less sensitive to noise and outliers, albeit at the cost of poorer model interpretability and more complex parameter tuning.

A robust model should perform well on both the calibration and prediction sets, avoiding either overfitting (excellent calibration metrics but poor prediction performance) or underfitting. The final criteria for evaluating model quality were the coefficient of determination for calibration (Rc^2^), the RMSECV, the coefficient of determination for prediction (Rp^2^), the RMSEP, and the Residual Predictive Deviation (RPD). An Rc^2^ closer to 1 and a lower RMSECV indicate a better calibrated model. Similarly, an Rp^2^ closer to 1 and a lower RMSEP signify stronger predictive ability and higher accuracy of the model. RPD, defined as the ratio of the standard deviation of the reference values to the RMSEP, serves as a key indicator of model robustness. A model is generally considered unreliable for practical application if RPD < 2. An RPD between 2.0 and 3.0 suggests the model is suitable for approximate quantification or good discrimination. An RPD > 3.0 indicates the model possesses excellent predictive capability and can be used for precise quantitative analysis.

All preprocessing, feature selection, and parameter tuning (including cross-validation) were performed exclusively on the calibration set. The test set was held out and used only once for final model evaluation to avoid data leakage.

## 3. Results and Discussion

### 3.1. Sample Set Partitioning

Rational and effective partitioning of the sample set is crucial for enhancing predictive accuracy and plays a vital role in model development. Prior to modeling analysis, the complete sample set needs to be divided into a calibration set and a prediction set according to a specific ratio. The calibration set is used to establish the model, while the prediction set is employed to assess the practical performance of the model. The method of sample set partitioning significantly influences the model outcomes. Ideally, the samples in the calibration set should encompass as wide range of the data space as possible. In this study, the Sample set Partitioning based on joint X-Y distances (SPXY) algorithm was employed for this partitioning. Unlike traditional methods that considering only spectral (X) or property (Y) distances, the SPXY algorithm incorporates variations in both spectral variables (X) and target chemical values (Y). This dual consideration facilitates the selection of samples that are uniformly distributed across the multidimensional data space [24]. Consequently, this method provides a more comprehensive representation of sample characteristics, thereby improving the predictive accuracy and robustness of the developed models. Therefore, the SPXY algorithm was adopted in this study to partition the sample set.

To evaluate the internal robustness of the model, 5-fold cross-validation was performed on the calibration set. The calibration set was randomly divided into five subsets of approximately equal size; each subset was used once as validation data while the remaining four subsets served as training data. The cross-validated coefficient of determination (R^2^cv) and root mean square error of cross-validation (RMSECV) were calculated as the average of the five iterations.

Based on repeated measurements of 10 samples, the pooled standard deviation of the oven-drying method was 0.031%. Accordingly, the expanded uncertainty was 0.062%. This uncertainty is substantially smaller than the overall standard deviation of the sample set (2.5%), indicating that the reference method possesses sufficient precision.

**Figure 1:**
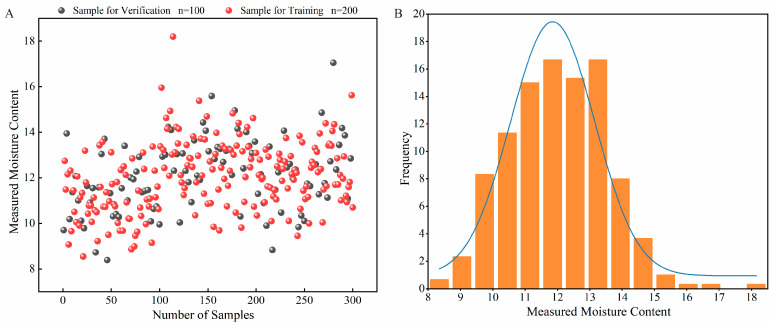
Scatters and normal distribution of Moisture content. (**A**) Hydrogen content distribution chart; (**B**) Normal distribution graph of moisture content.

Based on the SPXY algorithm, the samples were divided into a calibration set of 200 samples and a validation set of 100 samples, as shown in Figure 1A. The moisture content of all samples ranged from 8.3845% to 18.1973%. Specifically, the moisture content of the calibration set samples ranged from 8.5427% to 18.1973%, while that of the validation set ranged from 8.3845% to 17.0507%. The moisture content of the samples exhibited a wide distribution. Furthermore, as can be seen from Figure 1B, the data followed a normal distribution. This indicates that the areca nut samples are highly representative and exhibit significant variability, thus making them well-suited for the development of a near-infrared spectroscopy analysis model. The moisture content of the validation set samples was similar to that of the calibration set, suggesting that the samples selected for the validation set were reasonable and appropriate for model validation.

### 3.2. Original Spectra of Dried Areca Nuts and Spectral Preprocessing

**Figure 2:**
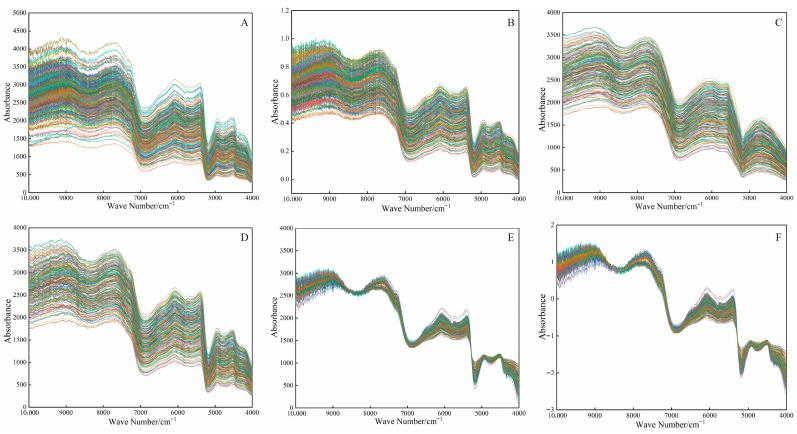
Near-infrared spectra of dried areca nuts, original spectrum (**A**), Normalized (**B**), SGS (**C**), airPLS (**D**), SNV (**E**), MSC (**F**).

The original near-infrared diffuse reflectance spectra of 300 dried areca nut samples are shown in Figure 2A. The spectra exhibit multiple absorption peaks within the 10,000^−1^–4000 cm^−1^ range. Although the general spectral patterns are consistent across the samples, the spectra are not perfectly superimposed, indicating their good reproducibility while also reflecting the intrinsic variability among the samples. The average relative standard deviation (RSD) of the samples across the entire spectral region ranged from 1.1% to 1.8%, indicating the absence of outliers in the sample set and demonstrating good instrument stability. The discrepancies observed among the samples may be attributed to noise and operational factors, underscoring the necessity for spectral preprocessing.

Five preprocessing methods, airPLS, Normalization, SGS, SNV, and MSC were applied to the original spectra. The results are presented in Figure 2B–F, showing distinct differences among the spectra processed by different methods. PLSR models were established using the original spectra and the spectra after each preprocessing method. The results are summarized in Table 2. The data presented in the table indicates that the model based on the original spectra exhibited commendable performance, achieving a coefficient of determination for the Rp^2^ and a RPD of 0.8871 and 4.63, respectively, demonstrating its substantial quantitative prediction capability. However, both airPLS and SGS led to a decrease in model performance to varying degrees, suggesting these methods are unsuitable for the moisture determination system in this study. In contrast, the application of MSC and SNV preprocessing techniques significantly improved the performance of the model. Notably, the model based on MSC preprocessed spectra yielded the best results. It achieved high coefficients of determination for both the calibration and prediction sets (Rc^2^ = 0.9964, Rp^2^ = 0.9639), a low RMSEP of 0.1960, and a markedly higher RPD value of 10.35 when compared to the model based on raw spectra.

These results strongly indicate that the near-infrared spectral signals related to moisture in the samples were severely affected by scattering effects. Both MSC and SNV effectively eliminated this background noise caused by physical factors, thereby enhancing the chemical information closely associated with moisture content. Consequently, MSC preprocessing was adopted for all subsequent modeling to ensure model stability and accuracy.

### 3.3. Modeling Results of Feature Wavelength Optimization Methods

#### 3.3.1. Feature Wavelength Selection Based on CARS

**Figure 3:**
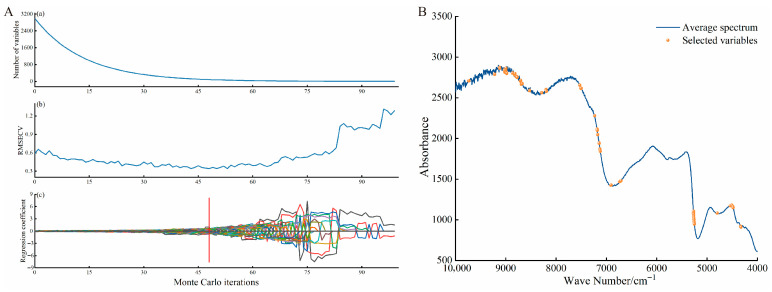
The CARS feature selection process and the distribution map of the selected features. (**A**(**a**)–**A**(**c**)) feature selection process; (**B**) Distribution map of features.

Feature wavelengths were screened using the CARS algorithm. To ensure the stability and representativeness of the feature selection, the Monte Carlo iterative sampling method was employed with the sampling run set to 100 times (Figure 3). Figure 3A comprises three panels, (a), (b), and (c), which respectively illustrate the dynamic changes in the number of feature wavelengths, the RMSECV, and the regression coefficient paths as the sampling runs increased. The vertical red dashed line in panel (c) marks the position corresponding to the minimum RMSECV. The optimal sampling run was determined to be the 48th, where the corresponding RMSECV value was the smallest. At this point, the number of selected characteristic wavelengths was 108. Their specific spectral positions are shown in Figure 3B.

#### 3.3.2. Feature Wavelength Selection Based on SPA

**Figure 4:**
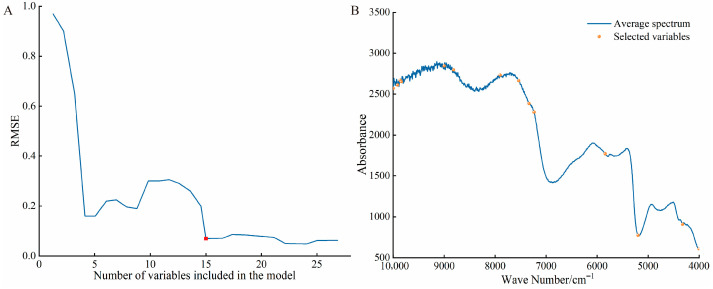
The SPA feature selection process and the distribution map of the selected features. (**A**) feature selection process; (**B**) Distribution map of features.

Feature wavelength screening was conducted on the MSC preprocessed NIR spectra using the SPA method. The optimal combination of characteristic wavelengths was determined by analyzing the trend of the Root Mean Square Error (RMSE) of the prediction set as the number of variables increased. As shown in Figure 4A, the model’s RMSE showed a decreasing trend when the number of variables increased from 0 to 15. The minimum RMSE (0.5032) was achieved when the number of variables was 15. Beyond this point, further increasing the number of variables resulted in no significant reduction in RMSE. This finding indicates that the 15-variable subset selected by SPA optimally balances feature dimensionality reduction with model accuracy. Consequently, the optimal number of variables was determined to be 15, with the specific spectral positions of these selected wavelengths detailed in Figure 4B.

#### 3.3.3. Stability of Feature Selection

To assess the robustness of the CARS feature selection, the selection frequency of each wavelength across the 100 Monte Carlo sampling runs was analyzed. The 108 wavelengths selected in the optimal run (RMSECV minimum) exhibited an average selection frequency of 0.72, with individual frequencies ranging from 0.55 to 0.98. These high frequencies indicate that the selected wavelengths were consistently identified across multiple random samplings, confirming the stability of the CARS algorithm. For the SPA method, the stability of the selected 15 wavelengths was evaluated by 5-fold cross-validation using the PLSR model. The cross-validation results (R^2^cv = 0.8237, RMSECV = 0.4389) were consistent with the independent test set performance (Rp^2^ = 0.8322, RMSEP = 0.4225), demonstrating that the SPA-selected feature subset provides stable and reliable predictions.

### 3.4. Establishment and Analysis of Quantitative Models

To systematically compare the effectiveness of the CARS and SPA feature wavelength selection methods and evaluate the performance of PLSR and SVR models, this study constructed PLSR and SVR models based on the variables selected by CARS and SPA, respectively. A full wavelength model served as a reference for comparison. All models aimed to predict moisture content, and the results are summarized in Table 3.

Our data reveals that the full wavelength PLSR model demonstrated superior predictive performance compared to the PLSR and SVR models built with CARS or SPA selected features. This phenomenon may be attributed to the following reasons. Firstly, during the process of eliminating redundant information, the CARS and SPA might have excessively removed spectral variables that have weak yet broad correlations with the target property, leading to the loss of effective information. Secondly, the absorption of water molecules in the near-infrared region primarily arises from the overtone and combination bands of O-H bonds. The main absorption peaks of water (e.g., around 5150 cm^−1^ and 6900 cm^−1^) are very broad spectral bands. This implies that moisture information is not concentrated at a few isolated wavelength points but is distributed diffusely across a wide spectral range. Furthermore, interactions between water and the sample matrix (such as proteins, cellulose, etc.) and the formation of hydrogen bonds subtly influence the shape, width, and position of the entire O-H absorption band. These subtle variations contain crucial information about the state of water in the sample, which requires the complete spectral data of the entire band and its surrounding regions to be fully captured. Feature selection can disrupt this synergistic network of information. Therefore, from the perspective of information integrity, for this specific dataset, the full wavelength model maximally retains these distributed, broad band details, thereby establishing a more accurate and robust prediction model.

For the optimal PLSR model based on full-wavelength MSC spectra, 5-fold cross-validation on the calibration set yielded an R^2^cv of 0.9592, an RMSECV of 0.2021, and an RPD is 10.04. These results, together with the independent test set metrics (Rp^2^ = 0.9639, RMSEP = 0.1960). The RPD value of 10.04 is comparable to the independent test set RPD of 10.35, confirming that the model is not overfitted and that its high predictive performance is stable across different data subsets, confirm the model’s excellent predictive stability and generalization capability.

### 3.5. Model Validation Using Independent Samples

**Figure 5:**
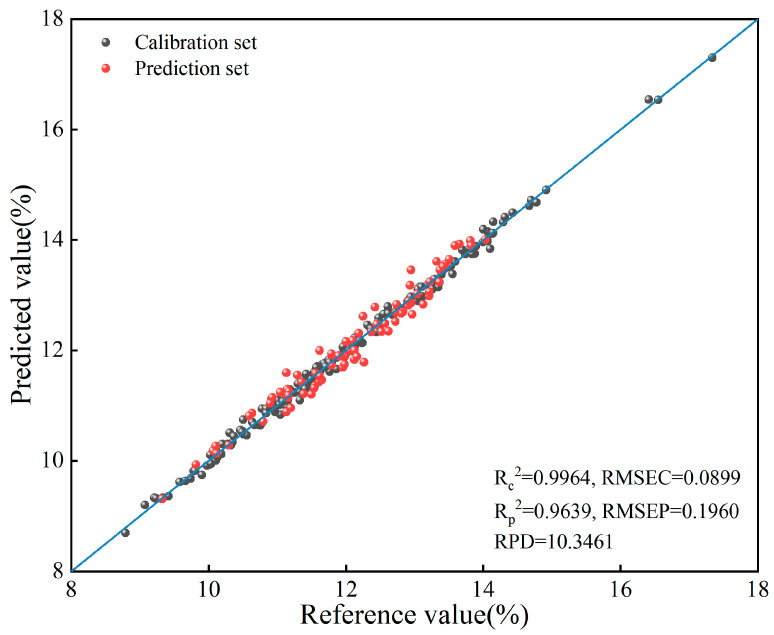
Regression graph of true values and predicted values.

To rigorously evaluate the robustness and reliability of the two simplified SOA-SVR models, an independent test set consisting of 100 areca nut samples was used for model assessment. The predictive performance of PLSR (Rp^2^ = 0.9639, RMSEP = 0.1960, RPD = 10.3461) was significantly better than that of SVR (Rp^2^ = 0.7119, RMSEP = 0.5537, RPD = 2.1075). Consequently, the PLSR model based on the full wavelength spectral data after MSC preprocessing was identified as the optimal choice for predicting the moisture content of dried areca nuts in this study. Figure 5 illustrates a scatter plot depicting the relationship between the predicted and observed values for the established model. The data points are relatively symmetrically distributed on both sides of the trend line, and the slope of the fitted curve is close to 1. This observation indicates a robust correlation and confirms the model’s good predictive performance.

**Figure 6:**
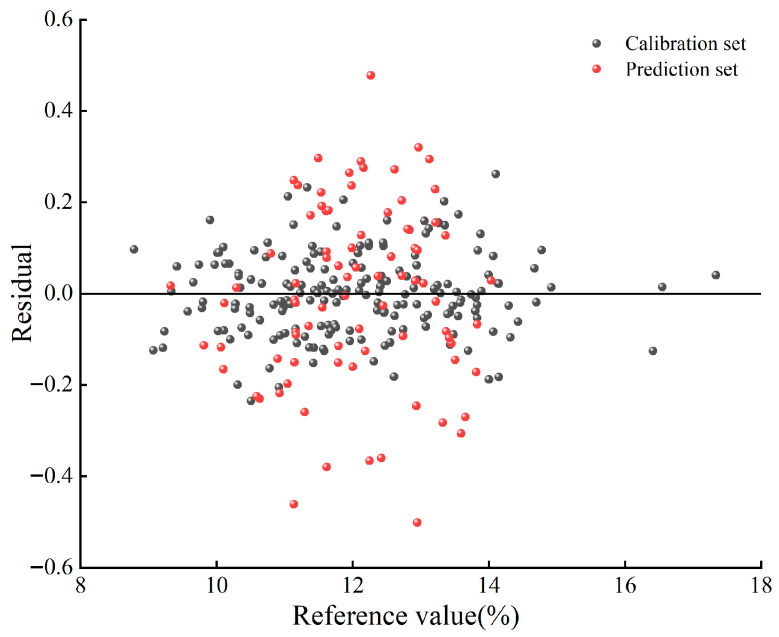
The residual plot of the sample.

To examine whether the optimal model exhibited any systematic prediction bias, a residual plot was generated for the test set samples (Figure 6). In this plot, the measured moisture content is displayed on the x-axis, and the residuals are displayed on the y-axis. As shown in Figure 6, the residual points are randomly distributed around the zero line without any discernible trend or pattern. This indicates that the model demonstrates good unbiasedness and stability, with no significant systematic errors detected. The Shapiro–Wilk test yielded a *p*-value of 0.137 (>0.05), indicating that the residuals follow a normal distribution, thereby satisfying the fundamental assumptions of linear regression. Additionally, the mean residual was −0.0036%, The 95% confidence interval is from −0.387% to 0.380%, which is close to zero, further confirming the unbiasedness of the model.

### 3.6. Paired t-Test of Samples

To statistically validate the precision and stability of the model, a paired *t*-test was conducted between the predicted moisture content values and the chemically measured values for the validation sample set. The results are presented in Table 4. The two-tailed *p*-value from the *t*-test was 0.98, which is greater than 0.05. This indicates that there is no statistically significant difference between the predicted and chemically measured moisture content values in areca nuts, there is no systematic deviation between the predicted values and the measured values.

## 4. Conclusions and Discussion

This study successfully established and validated a rapid, non-destructive method based on NIRS for the determination of moisture content in dried areca nuts. By employing a systematic evaluation of five spectral preprocessing methods, two feature wavelength selection algorithms, and a sample set partitioning method (SPXY), both PLSR and SVR models were constructed and compared. The optimal analytical pathway for each component was identified: the MSC-Full-PLSR strategy proved best for predicting moisture content. Optimal models exhibited excellent predictive capability, with the highest coefficient of determination for Rp^2^ reaching 0.9639 and the highest RPD reaching 10.3461, fully confirming the reliability and high accuracy of the method. An intriguing observation was that our data revealed that the full-wavelength PLSR model substantially outperformed the CARS-PLSR and SPA-PLSR models. This performance gap can be explained by the distinctive physicochemical properties of water in biological matrices. Water molecules form hydrogen bonds with cell wall components (cellulose, hemicellulose) and soluble solids (polyphenols, alkaloids), which broadens the O-H overtone and combination bands into continuous regions spanning. These broad bands encode information about different water states (free vs. bound) across many adjacent wavelengths. Feature selection algorithms such as CARS and SPA are designed to identify a minimal set of isolated, highly influential wavelengths; therefore, when applied to such broadband features, they inevitably discard neighboring correlated variables that carry complementary information. In contrast, the full-spectrum model retains the entire continuous regions and can exploit the collective predictive power of all wavelengths. This interpretation is consistent with established knowledge in near-infrared spectroscopy: for constituents with intrinsically broad absorption features (e.g., water, cellulose), full-spectrum or moderately reduced models often outperform aggressive variable selection. Therefore, we conclude that for moisture prediction in dried areca nuts, the full-wavelength PLSR model is both empirically superior and theoretically justified.

To understand why PLSR substantially outperformed SVR in this study, we examined the linearity of the spectral–moisture relationship and the intrinsic spectral properties of water. Pearson correlation coefficients were calculated between the MSC-preprocessed absorbance values and the reference moisture content at each wavelength across the calibration set. Across the entire spectral range (4000–10,000 cm^−1^), more than 70% of the wavelengths showed absolute correlation coefficients exceeding 0.35. However, high correlation alone does not prove linearity. Therefore, we performed a residual analysis on the optimal PLSR model (Figure 6). In contrast, the residuals in Figure 6 are randomly scattered around zero with no discernible pattern. The Shapiro–Wilk test confirmed normality (*p* = 0.137), and the mean residual (−0.0036%) was not significantly different from zero (paired *t*-test, *p* = 0.98). These residual diagnostics provide strong evidence that the linear PLSR model adequately captures the systematic variation without substantial lack-of-fit. These findings demonstrate a predominantly linear relationship between the spectral response and moisture content in this dataset. Therefore consistent with the linear nature of the data and the spectral characteristics of water.

This study introduces an optimized NIRS model tailored for the swift determination of moisture content in dried areca nuts. The proposed MSC-Full-PLSR methodology effectively overcomes the critical limitations of traditional oven drying methods, such as time consumption and destructiveness. It enables a reduction in analysis duration from several hours to just seconds, eliminating the need for sample preparation. This innovation serves as a robust instrument for potential online, real-time moisture monitoring in areca nut processing lines. This advancement is crucial for enhancing product quality control, optimizing drying processes, and reducing economic losses in the industry.

Although the developed model demonstrates remarkable efficacy, it is essential to recognize certain constraints that will inform investigations. Therefore, future work will focus on: expanding the sample library to enhance model universality, At the same time, additional external data validation is carried out for samples from different batches and using different instruments; integrating the calibration model into portable or inline NIRS systems for field and factory validation; and exploring the extension of this rapid detection framework to other critical quality indicators of areca nuts, such as total alkaloids or crude fiber content, to promote comprehensive quality control in the industry.

## Figures and Tables

**Table 1:** SVR optimization parameters.

Preprocess Method	C	γ	ε
MSC-FULL	100	scale	0.1
MSC-SPA	10	scale	0.1
MSC-CARS	10	scale	0.1

**Table 2:** Results of PLS Models Developed Using Different Pretreatment Methods.

Indicators	Preprocessing	Rc^2^	RMSEC	Rp^2^	RMSEP	RPD
Moisture Content	Raw	0.9675	0.2683	0.8871	0.3466	4.6333
	airPLS	0.8714	0.5341	0.7473	0.5186	2.5785
	Normalized	0.9675	0.2683	0.8871	0.3466	4.6333
	SGS	0.9406	0.3629	0.8720	0.3691	3.7416
	SNV	0.9959	0.0949	0.9598	0.2069	9.8018
	MSC	0.9964	0.0899	0.9639	0.1960	10.3461

**Table 3:** The Results of PLSR and SVR Models Developed Using Different Wavelength Selection Methods.

Wavelength Selection	Model	Rc^2^	RMSEC	Rp^2^	RMSEP	RPD
Full	PLSR	0.9964	0.0899	0.9639	0.1960	10.3461
	SVR	0.7883	0.6853	0.7119	0.5537	2.1075
CARS	PLSR	0.9264	0.4039	0.8517	0.3972	3.3965
	SVR	0.8395	0.5966	0.7214	0.5445	2.3489
SPA	PLSR	0.8949	0.4828	0.8322	0.4225	2.9349
	SVR	0.8454	0.5855	0.6538	0.6070	2.3050

**Table 4:** *t*-test.

Indicators	*t*	df	*p*	Mean Deviation
Moisture Content	−0.025	198	0.98	−0.00364

## Data Availability

The original contributions presented in the study are included in the article, further inquiries can be directed to the corresponding author.

## Abbreviations

The following abbreviations are used in this manuscript:

NIRS	Near-Infrared Spectroscopy
SNV	Standard Normal Variate
airPLS	adaptive iteratively reweighted Penalized Least Squares
MSC	Multiplicative Scatter Correction
SGS	Savitzky–Golay Smoothing
CARS	Competitive Adaptive Reweighted Sampling
SPA	Successive Projections Algorithm
PLSR	Partial Least Squares Regression
SVR	Support Vector Regression
SPXY	Sample set Partitioning based on joint X-Y distances
RMSECV	root mean square error of cross validation
RMSEP	root mean square error of prediction
Rp^2^	determination for prediction
Rc^2^	determination for calibration

## References

[1] Sun H., Yu W., Li H., Hu X., Wang X. (2024). Bioactive Components of Areca Nut: An Overview of Their Positive Impacts Targeting Different Organs. Nutrients.

[2] Zhang P., Chua N.Q., Dang S., Davis A., Chong K.W., Prime S.S., Cirillo N. (2022). Molecular Mechanisms of Malignant Transformation of Oral Submucous Fibrosis by Different Betel Quid Constituents—Does Fibroblast Senescence Play a Role?. Int. J. Mol. Sci..

[3] Aweau K.i., Erari S., Im S., Murphy K., Pokhrel P., Herzog T.A. (2024). What Motivates Betel Quid Chewers to Quit? An Analysis of Several Cessation-Relevant Variables. Subst. Use Misuse.

[4] Zhang J., Feng X., Xie S., Zhong Y., Sun Y., Wang W. (2025). Research on drying kinetics, microstructure, and flavor changes of areca nut drying process based on water molecule migration. Food Chem. X.

[5] Zhu M., Li J., Li X., Yao Y., Xu X., Hayat K., Tian F., Zhang X., Ho C.-T. (2025). Dynamic changes in fungal composition during the processing of Hainan areca nut and effects of water activity on dominant mold species. Int. J. Food Microbiol..

[6] Li M., Pang X., Gu Z., Guo Z., Xin Y., Zhang L. (2023). Rapidly analyzing of ingredients during chewing and processing of areca nut using feature-based molecular networking. Food Chem..

[7] Dai J., Kang X., Zhang J., Dai W., Wang Y., Sun Y., Wang Y., Qin H., Ji J., Wang S. (2025). Effect of electron beam irradiation treatment on microstructure, physicochemical properties, and bioactive content of areca nut. J. Sci. Food Agric..

[8] Yang B., Chen H., Chen W., Chen W., Zhong Q., Zhang M., Pei J. (2023). Edible Quality Analysis of Different Areca Nuts: Compositions, Texture Characteristics and Flavor Release Behaviors. Foods.

[9] Sagar V.R., Suresh Kumar P. (2010). Recent advances in drying and dehydration of fruits and vegetables: A review. J. Food Sci. Technol..

[10] Zhang M., Chen H., Mujumdar A.S., Tang J., Miao S., Wang Y. (2017). Recent developments in high-quality drying of vegetables, fruits, and aquatic products. Crit. Rev. Food Sci. Nutr..

[11] Huang Y., Bian Z., Jin H., Zheng G., Zhang Q., Hu D., Xie W., Fan C. (2026). Identification of early bruising degrees in blueberries using visible and near-infrared spectroscopy coupled with deep learning. Spectrochim. Acta Part A Mol. Biomol. Spectrosc..

[12] Moll V., Beć K.B., Handle L., Huck C.W. (2025). Wavelength-specific information in benchtop and handheld Vis-NIR and UV–Vis spectroscopy: The case of Coffea arabica. Food Res. Int..

[13] Li Z., Song J., Yang J., Tian H., Tang G., Lv Y., Zhang X., Li M. (2026). Quantitative prediction of active components in turnip using near-infrared spectroscopy (NIRS) and a multimodal feature fusion method. Spectrochim. Acta Part A Mol. Biomol. Spectrosc..

[14] Birenboim M., Kenigsbuch D., Shimshoni J.A. (2026). Rapid and robust quantification of ten cannabinoids in cannabis oils using FT-NIR spectroscopy coupled with PLS-R models. Spectrochim. Acta Part A Mol. Biomol. Spectrosc..

[15] Zhong Y., Li J., Liu H., Wang Y. (2025). Prediction of critical volatile organic compounds and identification of flavor intensity in Gastrodia elata under different preparation methods based on NIR and FTIR feature variables. Food Res. Int..

[16] Teye E., Amuah C.L.Y., Yeh T.-S., Nyorkeh R. (2023). Nondestructive Detection of Moisture Content in Palm Oil by Using Portable Vibrational Spectroscopy and Optimal Prediction Algorithms. J. Anal. Methods Chem..

[17] Cui H., Gu F., Qin J., Li Z., Zhang Y., Guo Q., Wang Q. (2024). Assessment of Peanut Protein Powder Quality by Near-Infrared Spectroscopy and Generalized Regression Neural Network-Based Approach. Foods.

[18] Sui Y., Zhao X., Ding J., Sun S., Tong Y., Ma W., Zhao Y. (2025). A nondestructive and rapid method for in situ measurement of crude fat content in soybean grains. Food Chem..

[19] Liu H., Chen F., Zhang L., Meng D., Sun H. (2025). Improvement method for tea leaf moisture content prediction using VIS-NIR spectrum based on transfer learning. Spectrochim. Acta Part A Mol. Biomol. Spectrosc..

[20] Ordoñez-Lozano S., Collazos-Escobar G.A., Bahamón-Monje A.F., Gutiérrez-Guzmán N. (2025). Monitoring moisture content in parchment coffee beans during drying using Fourier Transform near infrared (FT-NIR) spectroscopy: A dataset for calibrating chemometric-based models for moisture prediction. Data Brief.

[21] Li X., Xu Z., Tang L., Zhao G., Wu Y., Zhang P., Wang Q. (2024). An effective moisture interference correction method for maize powder NIR spectra analysis. Spectrochim. Acta Part A Mol. Biomol. Spectrosc..

[22] Biswas A., Chaudhari S.R. (2024). Exploring the role of NIR spectroscopy in quantifying and verifying honey authenticity: A review. Food Chem..

[23] Malavi D., Raes K., Van Haute S. (2024). Integrating near-infrared hyperspectral imaging with machine learning and feature selection: Detecting adulteration of extra-virgin olive oil with lower-grade olive oils and hazelnut oil. Curr. Res. Food Sci..

[24] Dai J., Tang W., Zhang J., Kang X., Dai W., Ji J., Wang S. (2024). Determination and quality evaluation of active ingredients in areca nut using near-infrared rapid detection technology. Microchem. J..

[25] Zhang Y., Wu W., Zhou X., Cheng J.-H. (2025). Non-Destructive Detection of Soybean Storage Quality Using Hyperspectral Imaging Technology. Molecules.

[26] Yin H., Mo W., Li L., Ma Y., Chen J., Zhu S., Zhao T. (2024). Near-Infrared Spectroscopy Analysis of the Phytic Acid Content in Fuzzy Cottonseed Based on Machine Learning Algorithms. Foods.

[27] Wu C., Feng Y., Cui J., Yao Z., Xu H., Wang S. (2025). Detection of Quality Deterioration of Packaged Raw Beef Based on Hyperspectral Technology. Food Sci. Nutr..

[28] Li X., Wei Z., Peng F., Liu J., Han G. (2023). Non-destructive prediction and visualization of anthocyanin content in mulberry fruits using hyperspectral imaging. Front. Plant Sci..

[29] Li H., Jia S., Le Z. (2019). Quantitative Analysis of Soil Total Nitrogen Using Hyperspectral Imaging Technology with Extreme Learning Machine. Sensors.

[30] Yu Z., Chen X., Zhang J., Su Q., Wang K., Liu W. (2023). Rapid and Non-Destructive Estimation of Moisture Content in Caragana Korshinskii Pellet Feed Using Hyperspectral Imaging. Sensors.

